# Effects of Disinfection Methods on the Color Stability of Precolored and Hand-Colored Maxillofacial Silicone: An In Vitro Study

**DOI:** 10.1155/2022/7744744

**Published:** 2022-06-13

**Authors:** Natdhanai Chotprasert, Binit Shrestha, Kawin Sipiyaruk

**Affiliations:** ^1^Maxillofacial Prosthetic Clinic, Department of Prosthodontics, Faculty of Dentistry, Mahidol University, Bangkok, Thailand; ^2^Department of Orthodontics, Faculty of Dentistry, Mahidol University, Bangkok, Thailand

## Abstract

Maxillofacial prostheses are used in rehabilitation of patients with facial defects. Typically, these prostheses are fabricated with medical grade silicone and are tinted corresponding to the patients' natural skin color. However, exposure to environment and disinfectants can result in color changes. This study aimed to evaluate the effects of four different disinfection methods on the color stability of precolored and hand-colored maxillofacial silicones. Forty specimens each of precolored and hand-colored silicone were prepared. The specimens were randomly divided into eight groups (*n* = 10) and cleansed with four different disinfection methods. Disinfection was carried out six times/day for 60 days, simulating once-a-day disinfection for a year. Color evaluation was carried out at day 0 and day 60 using a UV-vis spectrophotometer. Color alterations were calculated by the CIE *L*^*∗*^*a*^*∗*^*b*^*∗*^ system. Data were analyzed by two-way ANOVA with post hoc Tukey HSD and *t*-tests (*α* = 0.05). Disinfectants can affect the color stability of maxillofacial silicone. In our study, chlorhexidine solution and liquid soap resulted in the highest color change. Precolored silicone showed higher color stability than its hand-colored counterpart.

## 1. Introduction

Maxillofacial prostheses are extensively used in the aesthetic rehabilitation of patients with facial defects. Silicone elastomer is the most common material due to its physical properties, such as good strength, durability, flexibility, skin-like texture, and acceptable biocompatibility [1, 2]. Furthermore, it can also be colored intrinsically and extrinsically to confer to the patient's skin color greatly improving aesthetics and acceptance by maxillofacial prosthodontists and patients alike [3]. Intrinsic coloring of silicone can be attained by the use of precolored silicone, which are available in different shades as made available by the manufacturer. It is also possible to manually incorporate different oil, powder, or silicone-based colors of various shades to a transparent silicone base, a procedure referred as hand-coloring. At times, both techniques can also be combined to attain the desired shade. Factors such as the production method of color, homogeneity of dispersion, and its ability to physically or chemically integrate with the silicone network can affect how the color pigments interact with the silicone network and its stability over time [3].

The discoloration of silicone is one of the primary disadvantages and can severely limit the shelf-life of the prosthesis. It occurs following exposure to external factors such as UV light, air pollution, cosmetics, temperature changes, humidity, and the use of various disinfection procedures due to the highly permeable nature of silicone [4–6]. Although many cleansing agents have been recommended, including water, neutral soap, and chlorhexidine [7], they should be used carefully as they can negatively affect the physical properties of the material [8]. Moreover, different methods of intrinsic coloring can also be one of the influencing factors for the color stability of silicone [9].

To the best of our knowledge, there had been no study on the effects of disinfectant on the color stability of precolored and hand-colored silicone. The objectives of this study were to evaluate the effects of intrinsic coloring techniques and disinfection methods on the color stability of maxillofacial silicone after a simulated 1-year period of cleansing, using the CIELAB color system. The understanding achieved from this research would support maxillofacial prosthodontic education, research, and practice in the selection of appropriate disinfection agents or methods to minimize color alteration of maxillofacial silicone.

## 2. Materials and Methods

For the precolored and hand-colored groups, medical grade silicone (country shade, Multisil Epithetik; Bredent Inc, Germany) and colorless transparent silicone (Multisil Epithetik; Bredent Inc, Germany) were chosen, respectively. For each group, 40 specimens were prepared individually, and silicone base and catalyst were mixed in the ratio of 1 : 1 by weight as recommended by the manufacturer. A thixotropic agent (Thixo; Factor II, Inc, USA) was also added to the silicone mixture (2 drops per 10 g of silicone) to aid in manipulation. Additionally, for the hand-colored group, 2% weight of intrinsic silicone coloration pigments (Santa Fe shade, FI-SK 11; Factor II Inc, USA) was added to the mixture. After attaining a homogenous blend, the specimen was packed into a custom-made cylindrical stainless-steel mold (22 mm diameter × 3 mm thickness). The specimens were allowed to vulcanize for 12 hours in room temperature. Following which, the specimens were removed from the mold and visually checked for surface irregularities and internal defects. Specimens with visible voids and cracks were excluded from the study. Both groups represented the average Thai base skin tone and were visually indistinguishable from one another.

Eighty specimens from both groups were then randomly divided into 8 groups (*n* = 10) and treated with four different disinfection methods: P-DW, precolored silicone washed with distilled water (control); MM-DW, hand-colored color silicone washed with distilled water (control); P-CHX, precolored silicone disinfected with 2% chlorhexidine solution (MDent, Faculty of Dentistry, Mahidol University, Thailand); MM-CHX, hand-colored color silicone disinfected with 2% chlorhexidine solution; P-CHXS, precolored silicone disinfected with chlorhexidine liquid soap (Q-Bac 4, Pose Health Care, Thailand); MM-CHXS, hand-colored silicone disinfected with chlorhexidine liquid soap; P-AS, precolored silicone disinfected with antibacterial liquid soap (Dettol, PT. Reckitt Benckiser, Indonesia); MM-AS, hand-colored silicone disinfected with antibacterial liquid soap. The specimens were stored in a dark chamber at room temperature for 24 hours before measuring the color value using the UV-visible reflectance spectrophotometer (ColorFlex 45/0, HunterLab, USA) and CIE *L*^*∗*^*a*^*∗*^*b*^*∗*^ color system, *L*^*∗*^ for the lightness from black (0) to white (100), *a*^*∗*^ from green (-) to red (+), and *b*^*∗*^ from blue (-) to yellow (+). The results were recorded as day 0. Three measurements were made for each specimen, and mean value was considered.

For each disinfection cycle, specimen from P-DW and MM-DW groups were completely immersed in distilled water for five minutes at room temperature, whereas P-CHX and MM-CHX groups were immersed in 2% chlorhexidine gluconate solution for five minutes at room temperature. In P-CHXS and MM-CHXS groups, chlorhexidine liquid soap was hand rubbed on silicone specimens and P-AS and MM-AS groups with antibacterial liquid soap. The specimens in these four groups were hand rubbed for 30 seconds during the disinfection cycle.

Following disinfection, each specimen was rinsed thoroughly with running tap water for 30 seconds and dried with tissue paper. The cycle was sequentially repeated for six times/day, followed by storage in a dark chamber. Similarly, the disinfection cycles were daily repeated for a total of 60 days, simulating a 1-year period of disinfectant usage by the patients.

On day 60, the color value of each specimen was measured again using the UV spectrophotometer. All of the values were recorded in the CIE *L*^*∗*^*a*^*∗*^*b*^*∗*^ color system; the mean Δ*L*^*∗*^, Δ*a*^*∗*^, Δ*b*^*∗*^ value for each specimen was obtained to calculate the color difference (∆E):

(1) $$\begin{array}{c} \Delta E=\sqrt{{\left(\Delta L^{*}\right)}^{2}+{\left(\Delta a^{*}\right)}^{2}+{\left(\Delta b^{*}\right)}^{2}}. \end{array}$$

The null hypothesis was set as there would be no difference in the color stability outcomes of different types of intrinsically colored silicones following disinfection by different solutions.

The statistical analysis was performed using IBM SPSS statistic with a significance level of *α* = 0.05. The effect of silicone types and disinfection methods on color stability was analyzed by two-way analysis of variance (ANOVA). A post-hoc test (Tukey HSD) was used to analyze the color change on different disinfection methods regardless of the type of silicone. In addition, regardless of the disinfection methods, the effect of different types of silicones on color change was evaluated by the *t*-test.

## 3. Results

The present study rejected the null hypothesis that there would be no difference in the color stability outcomes of different types of intrinsically colored silicones following disinfection by different solutions.

**Figure 1:**
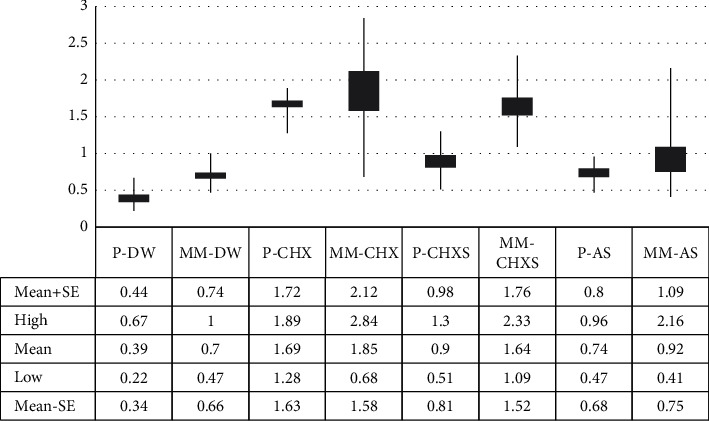
Color change (∆E) associated with precolored and hand-colored silicone after 1 year of stimulated disinfectant usage. DW, distilled water; CHX, 2% chlorhexidine solution; CHXS, chlorhexidine liquid soap; AS, antiseptic liquid soap.

The mean with standard error of color stability in each type of intrinsically colored silicone after 1-year of simulated disinfection is shown in Figure 1. The highest color change was observed in the MM-CHX group (1.85 ± 0.27), and the lowest color change was found in the P-DW group (0.39 ± 0.05), with a statistically significant difference between the two combinations (*p* < 0.001). Two-way ANOVA showed that two main factors (types of silicone and disinfection methods), significantly affected color stability (*F* = 2.47, *p* < 0.001, and *F* = 17.11, *p* < 0.001, respectively). However, the interaction between the type of intrinsically colored silicone and disinfection method did not significantly affect the color stability (*F* = 1.11, *p* = 0.093), as given in Table 1.

Regardless of the type of silicone, the highest color change was found following disinfection with 2% chlorhexidine solution (1.77 ± 0.13), followed by chlorhexidine liquid soap (1.27 ± 0.11), antibacterial soap (0.83 ± 0.09), and the least with distilled water (0.55 ± 0.05). A post-hoc test (Tukey HSD test) revealed that the color change of silicone after disinfectant with 2% chlorhexidine solution was significantly higher than distilled water (*p* < 0.001), antibacterial soap (*p* < 0.001), and chlorhexidine liquid soap (*p* = 0.001). However, the color stability following disinfection with antibacterial soap was not significantly different than distilled water (*p* = 0.136), as shown in Table 2.

Regardless of the disinfection methods, the *t*-test indicated that the hand-colored silicone group (1.28 ± 0.11) had significantly higher color change than the precolored silicone group (0.93 ± 0.08) (*p*=0.014), as given in Table 3.

## 4. Discussion

Silicone prosthesis generally has a lifespan for 1.5–2 years [10]. One of the common problems following its use is the discoloration of silicone. Several environmental factors such as solar radiation, humidity, temperature, and airborne pollutants, as well as routine cleaning, can induce color alterations of maxillofacial silicone prostheses [11]. The primary mechanism has been attributed to chemical alteration within the material due to UV radiation in combination with air and moisture. This leads to bond breakage within the silicone polymeric network and color pigments, leading to their alteration or disintegration. Lightfastness values of colors can vary depending on their origin. Inorganic colors are ionic bond-based metal oxide and have greater color stability than organic ones that tend to replace their double or triple bonds [12].

In this study, the effects of the intrinsic coloring technique (precolored and hand-colored) and different disinfection methods on color stability were evaluated. The silicone (Multisil Epithetik) used in this study was based on a single manufacturer (Bredent Inc., GmbH and Co. KG, Germany). The precolored silicone in country shade was chosen as it had the most resemblance with the average Thai base skin tone. To minimize bias, the hand-colored specimens were tinted to produce a similar color such that the two groups were visually indistinguishable from one another. All specimens, whether precolored or hand-colored, showed different amounts of color alterations after disinfection and were in accordance with previous literature [5, 13–16]. Table 1 demonstrates that both factors, intrinsic coloring techniques and disinfection methods, significantly affected the color stability of silicone.

Precolored silicone showed significantly higher color stability over hand-colored silicone regardless of the disinfection method. Higher color stability could be assumed due to the stable binding of the colors with silicone polymeric network. Furthermore, regardless of the coloring technique, 2% chlorhexidine solution showed significantly higher color alteration than other disinfection methods. Conversely, past studies on color stability of silicone have shown varied results. Chamaria et al. found that antibacterial soap (Dettol) had higher color change compared to 2% chlorhexidine solution [13]; whereas, Goiato et al. found that 4% chlorhexidine solution showed the highest color alteration followed by neutral soap and Efferdent [14]. These inconsistencies in color change among the studies may have been due to differences in specimen preparation, methodology, condition for exposure, active ingredients present in the disinfectant, and study duration. In our observations, 2% chlorhexidine solution had significantly higher color alteration followed by chlorhexidine liquid soap, antibacterial liquid soap, and distilled water.

A perceivable color difference may vary from one observer to another; thus, in this study, objective analysis of color difference was carried out using a spectrophotometer. Color differences in the range of 0-1 represents color identical to the reference and is unperceivable to the normal human eye, the range of 1-2 can be perceived as a color difference by experienced observers, and values > 3 can be considered as clinically unacceptable [13], especially during maxillofacial rehabilitation when aesthetics is of a primary concern [17]. At simulated 360 days, the mean color difference in all groups was slightly raised. Although the color changes associated with distilled water and antiseptic soap remained below the perceivable value of < 1 for both precolored and hand-colored silicone, the mean ∆E of MM-CHX, MM-CHXS, and P-CHX indicated considerable color changes which were enough to be detectable by experienced observers. Visually, these specimens appeared darker than the others. The highest mean ∆E of 1.85 was observed in the hand-colored group disinfected with 2% chlorhexidine gluconate. However, the value was still lower than the study by Chamaria et al. where pigmented silicone showed ∆*E* = 2.42 after exposure to 2% chlorhexidine and ∆*E* = 4.86 with antibacterial liquid soap (Dettol) [13]. These variations could have resulted due to the type of silicone [18] and intrinsic colors used [19], as Chamaria et al. utilized dry pigment, whereas this study utilized silicone pigment [13, 20]. Dry earth pigments bears morphological resemblance to cosmetic powders, and owing to their large size may only remain dispersed rather than incorporated in the polymer matrix, thus making them more susceptible to UV degradation [20]. According to the manufacturer of silicone pigment, Factor 2, it is a blend of cosmetic pigments crushed into a silicone cross-linking fluid and designed to chemically bind with the silicone polymeric network [21]. Even though the silicone pigments provided better color stability compared to dry pigments, the values were still lower than precolored silicone.

In our study, hand-colored silicone showed lesser color stability; however, clinicians frequently use it during prosthesis fabrication as the shade of precolored silicone may not completely match the patient's skin tone in a clinical setting and additional coloring may be needed. Furthermore, procedures such as hand rubbing of the disinfectants could also cause frictional wear and dislodgement of passively dispersed surface pigments, thus establishing the need for frequent restraining of the prosthesis. In order to minimize color changes, it is recommended to avoid strong disinfectants, such as chlorhexidine and use precolored silicone when possible. The addition of opacifiers or intrinsic UV light absorbers may also minimize color degradation, but their addition should be limited to reduce changes to the physical properties of silicone [12].

One of the limitations of the research was that all specimens could not be prepared simultaneously. This could have led to some errors in obtaining a homogenous dispersion of coloring pigments among the specimens, especially with the hand-coloring technique. Uneven distribution of color could inherently affect the color values. However, our study considered mean ΔE values which could have minimized this error. In addition, due to the product availability, our study investigated silicone products from a single manufacturer, and therefore, the results obtained may not be fully applicable for products from other manufacturers. Further studies on the development of agents or methods to minimize color alteration of maxillofacial silicone as well as on the comparison of silicone products from different manufacturers should be required to enhance our understanding of the material.

## 5. Conclusions

Chlorhexidine in both solution and liquid soap forms resulted in the highest color change in both types of intrinsically colored silicone compared to the other disinfectants. In addition, precolored silicone had greater color stability than hand-colored silicone. Further research on the development of agents or methods to minimize color alteration of maxillofacial silicone should be required to extend prosthesis life.

## Figures and Tables

**Table 1:** Two-way ANOVA results for color change (∆E) of silicone after stimulated 1-year disinfectant usage.

Source	Sum of squares	df	Mean squares	F	*P* value
Disinfection methods	17.11	3	5.70	34.27	<0.001
Type of silicones	2.47	1	2.48	14.86	<0.001
Interaction (disinfection methods *∗* type of silicones)	1.11	3	0.37	2.22	0.093
Error	11.99	72	0.17		

**Table 2:** Mean and standard error of color change (∆E) for four disinfection methods regardless of the type of intrinsically colored silicone.

Disinfectant	Mean	Standard error	*n*	Post hoc test (Tukey HSD test)	
				Pair	*P* value
Distilled water (DW)	0.55	0.05	20	DW/CHX	<0.001
2% chlorhexidine solution (CHX)	1.77	0.13	20	DW/CHXS	<0.001
Chlorhexidine liquid soap (CHXS)	1.27	0.11	20	DW/AS	0.136
Antibacterial liquid soap (AS)	0.83	0.09	20	CHX/CHXS	<0.001
				CHX/AS	<0.001
				CHXS/AS	0.006

**Table 3:** Mean and standard error of color change (∆E) for precolored and hand-colored silicone regardless of the disinfection method.

Type of silicone	Mean	Standard error	*n*	*P* value
Precolored	0.93	0.08	40	0.014
Hand-colored	1.28	0.11	40	

## Data Availability

The data used to support this study are available from the corresponding author upon request.
